# Prognostic relevance of topoisomerase II α and minichromosome maintenance protein 6 expression in colorectal cancer

**DOI:** 10.1186/s12885-019-5631-3

**Published:** 2019-05-09

**Authors:** A. Hendricks, F. Gieseler, S. Nazzal, J. H. Bräsen, R. Lucius, B. Sipos, J. H. Claasen, Th. Becker, S. Hinz, G. Burmeister, C. Schafmayer, C. Schrader

**Affiliations:** 1 Department of General and Thoracic Surgery, University Hospital Schleswig-Holstein, Campus Kiel, Arnold-Heller Str. 3, Hs. 18, 24105 Kiel, Germany; 2 First Department of Medicine, UKSH, Campus Lübeck, Lübeck, Germany; 3 Department of Medicine, Baruch Padeh Poria Medical Center, Faculty of Medicine in the Galilee, Bar-Ilan University, Tiberias, Lower Galilee Israel; 4 Institute of Pathology, Hannover Medical School, Hannover, Germany; 5 Institute of Anatomy, University of Kiel, Kiel, Germany; 6 Institute of Pathology, University of Tübingen, Tübingen, Germany; 7 Clinic of Forensic Psychiatry Nette-Gut, Weißenthurm, Germany; 8 Praxis Dr. Schrader, Kiel, Germany

**Keywords:** Colorectal cancer, Proliferative proteins, Minichromosome maintenance protein 6, Topoisomerase II α, Prognostic marker

## Abstract

**Background:**

Despite rising incidence rates of colorectal malignancies, only a few prognostic tools have been implemented in proven clinical routine. Cell division and proliferation play a significant role in malignancies. In terms of colorectal cancer, the impact of proliferation associated proteins is controversially debated. The aim of our study was to examine the expression of topoisomerase II α and minichromosome maintenance protein 6 and to correlate these findings with the clinical data.

**Methods:**

Tissue samples of 619 patients in total were stained using the antibodies Ki-S4 and Ki-MCM6 targeting topoisomerase II α as well as minichromosome maintenance protein 6. The median rate of proliferation was correlated with clinical and follow up data.

**Results:**

The expression rate of minichromosome maintenance protein 6 is significantly higher than the proportion of topoisomerase II α in tumour cells (*p* < 0.001). A high expression of both proteins coincides with a beneficial outcome for the patient, indicating a favourable prognostic marker (*p* < 0.001 and *p* = 0.008).

**Conclusions:**

We have demonstrated that high expression rates of proliferative markers is linked to a beneficial patient outcome. According to the general opinion, a high expression rate correlates with a poor patient outcome. In this study, we were able to refute this assertion.

**Electronic supplementary material:**

The online version of this article (10.1186/s12885-019-5631-3) contains supplementary material, which is available to authorized users.

## Background

Colorectal malignancies are a major cause of death in industrialised countries. Most colorectal neoplasms are histologically adenocarcinomas and develop through an adenoma-carcinoma sequence which was first described by Vogelstein and Fearon [1]. The development of a colorectal carcinoma depends on various factors and may often span over years before a manifest malignancy occurs. The macroscopic shape, histological type and grading seem to play key roles in the transformation process as defined by the adenoma-carcinoma sequence. Also, genetic mutations significantly affect the likelihood of colorectal cancer formation [2].

Mitosis within the neoplasia plays a key role in the histopathological analysis of the tumour. Assessment of the proliferation rate by means of proliferation markers is routinely implemented in histological diagnostics. Monoclonal antibodies against antigens associated with cell proliferation, such as Ki-67 [3, 4] and proliferating cell nuclear antigen (PCNA) are part of routine diagnosis in malignancies. Besides these mentioned proteins there are additional proliferation associated proteins, such as topoisomerase II α (Topo II α) and the minichromosome maintenance protein 6 (MCM6), that can be detected by immunohistochemistry (IHC) [5, 6]. The group of topoisomerases comprises up to four enzymes that are essential in the DNA topology and crucial for DNA replication [7]. By applying the monoclonal antibody Ki-S4, Topo II α can be detected by IHC [5, 8]. The prognostic significance of expressed Topo II α by Ki-S4 was shown in different studies [9–11]. High rates of expressed Topo II α correspond to an unfavourable clinical outcome. However, only a few studies comprising CRC patients have been published so far. Within these studies, fluorescent in situ hybridization (FISH) was applied to detect the expression rate of Topo II α. IHC has not been exerted to evaluate the clinical outcome of patients suffering from colorectal neoplasm yet.

Minichromosome maintenance proteins also play a key role in DNA replication of eukaryotic cells. These proteins are a part of the pre-replication complex, which binds to chromatin and therefore represent an essential role in cell division [12]. Ki-MCM6 is a specific antibody targeting MCM6 that can be used in formalin fixed tissue [6, 13, 14]. Multiple studies have verified the clinical relevance of MCM proteins as proliferation markers in malignant tumours so far [15–17]. Though, to the best of our knowledge, no investigation of the clinical relevance in terms of clinical outcome of MCM6 in colorectal carcinoma patients in a representative cohort has been published.

This publication aims to investigate the clinical relevance of topoisomerase II α and minichromosome maintenance protein 6 as proliferation markers in a representative large cohort of human colorectal carcinoma tissue. Results in terms of immunohistochemical expression are correlated to clinical follow-up data. Furthermore, it has to be investigated, whether the degree of expressed proliferation markers varies between clinical-pathological profiles.

## Methods

### Patients

A total of 619 patients was included in this study. All patients underwent a complete oncological resection of a histologically verified colorectal carcinoma at the Department of General and Thoracic Surgery, University Hospital Schleswig Holstein, Campus Kiel, during the period of 1994 and 2007. The resected tumour tissue was preserved at the Institute of Pathology, University Hospital Schleswig Holstein, Campus Kiel. Clinical and follow up data were gathered retrospectively. All data are shown in Table 1. The study was approved by the local ethics committee of the Medical Faculty, Christian-Albrechts University Kiel (reference no. A110/99).

**Table 1:** Patient demographics, clinical characteristics and univariate analysis (log rank test) influencing the overall survival (OS) disease free survival (DFS)

	N (%)	OS [months]	P	DFS [months]	P
all	619 (100)				
age (years)					
< 65	303 (48.9)	n.a.	**< 0.001**	59.5	**0.005**
≥ 65	315 (50.9)	65.6		n.a.	
unknown	1 (0.2)				
sex					
male	312 (50.4)	119.1	0.961	n.a.	0.218
female	307 (49.5)	104.3		n.a.	
tumor site					
right colon	172 (27.8)	130.5	**0.010**	n.a.	0.299
left colon + rectum	439 (70.1)	69.5		n.a.	
unknown	8 (1.3)				
UICC					
I + II	297 (48.0)	154.6	**< 0.001**	n.a.	**< 0.001**
III	199 (32.1)	87.0		49.8	
IV	117 (18.9)	22.1		13.7	
unknown	6 (1.0)				
histological grading					
I	10 (1.6)	n.a.	**< 0.001**	n.a.	**0.007**
II	505 (81.6)	122.4		n.a.	
III	102 (16.5)	41.8		33.6	
unknown	2 (0.3)				
histology					
adeno carcinoma	525 (84.9)	122.6	**< 0.001**	n.a.	**0.010**
mucinous carcinoma	74 (12.0)	68.3		40.7	
signet-ring cell carcinoma	7 (1.1)	12.3		9.4	
unknown	13 (2.1)				
resection margin					
R0	573 (92.6)	15.3	**< 0.001**	n.a.	**< 0.001**
R1 + R2	32 (5.2)	122.6		10.7	
unknown	14 (2.3)				
therapy					
sole surgical resection	229 (37.0)				
+ chemotherapy	136 (22.0)				
+ radiation	10 (1.6)				
+ chemoradiation	111 (17.9)				
+ unknown	133 (21.5)				

All *P* values in bold, are regarded as statistically significant. Abbreviations: *n.a.* not achieved, *UICC* Union internationale contre le cancer

### Immunohistochemistry

Formalin fixed tissue embedded in paraffin was cut into 3–5 μm thin slices using a microtome (Jung, Heidelberg, Germany). The sections were transferred to covered microscope slides (Histobond, Marienfeld, Germany) at a temperature of 45–55 °C. Before staining, all slides were applied to 100% xylol for 10 min to deparaffinise the tissue. For rehydration, all slides were transferred into a descending sequence of ethanol (100, 96, 70%) for 3 minutes each.

All sections were stained using haematoxylin-eosin stain. After rehydration, the sections were incubated with 200 μl haematoxylin for 10 min and rinsed with distilled water for 10 min. The sections were then incubated in 400 μl eosin for 3 min and rinsed with distilled water. Finally, all sections were applied to an ascending sequence of ethanol (70, 96, 100%) and subsequently incubated in xylol for 5 min.

### Analysis of immunohistochemical staining

All tissue sections were treated with highly specific monoclonal antibodies against the respective antigen and an indirect detection using a secondary antibody. Endogenous peroxidase was blocked by incubation of the specimens in 4 ml 30% hydrogen peroxide and 200 ml methanol. Antigen retrieval was performed by incubation in 0.01 M citrate buffer solution (pH 6.0) for 3 min at 100 °C [18]. In the next step, all sections were rinsed with water and transferred into washing buffer. All tissue samples were incubated with 100 μl of the primary antibody (detection of topoisomerase II α: Ki-S4; detection of minichromosome maintenance protein 6: Ki-MCM6; Institute for Haematopathology Kiel, University Hospital Schleswig Holstein, Campus Kiel) at room temperature for 60 min and afterwards incubated in tris-buffered saline (TBS), washed with water and then moved to TBS. The secondary antibody (Rabbit anti-mouse IgG; E354 DAKO, Hamburg, Germany) was applied at room temperature for 30 min. In the next step, slides were rinsed with water and transferred into washing buffer. The sections were stained with 100 μl DAB (Diaminobenzidin, DAKO, Hamburg, Germany) and rinsed twice with distilled water. Nucleus counter staining was achieved by hemalum (Merck, Darmstadt, Germany) incubation for 5 minutes. For dehydration purpose, all specimens were moved along an ascending incubational sequence of ethanol (70, 96 and 100%) and incubated twice in xylol.

The tissue specimens on microscopic slides were covered with Pertex (Medite, Burgdorf, Germany) and light microscopy was performed using the Axioskop 40 (Zeiss, Germany). Within each specimen 500 tumour cells in five randomly selected visual fields were examined using a cell counter (Counter AC8, Hecht AG, Sondheim, Germany) at a magnification of 400 times. Areas with exceptional high number of tumour cells were accounted separately as hot spots.

The primary antibodies Ki-S4 and Ki-MCM6 were established beforehand and the specificity was consolidated by Western blot experiments previously [8, 13].

### Statistical analysis

Comparative statistical analysis of expressed proliferation markers was performed using Fisher’s tests of significance. The univariate analysis of survival was done using the Log rank test and Kaplan-Meier analysis. The software GraphPad Prism, Version 7.0 (GraphPad Software, La Jolla, CA, USA) was used for statistical analysis. The significance level was set at 5% (*p* < 0.05).

## Results

### Patient cohort and clinical characteristics

The examined cohort consisted of 619 patients (50.4% male; 49.6% female). The median age was 65.2 years (mean 66 years; range 29 to 102 years). All considered clinical and histopathological characteristics, had a significant impact on the patient outcome in terms of overall survival (OS) and progression-free survival (PFS). However, other parameters like gender and tumour localisation did not have any effect on the outcome (Table 1). Patients aged ≥65 years had a significantly worse OS (*p* < 0.001) and PFS (*p* = 0.005). Staging by UICC displayed a significant effect on the OS (*p* < 0.001) and PFS (*p* < 0.001). Patients diagnosed with advanced tumours and local and/or remote metastasis (UICC III + IV) displayed a highly significant poorer outcome. In our view, the cohort represents the general population (Additional file 1: Figure S1 A + B).

### Expression of topoisomerase II α correlated to clinic-pathological characteristics

**Fig. 1:**
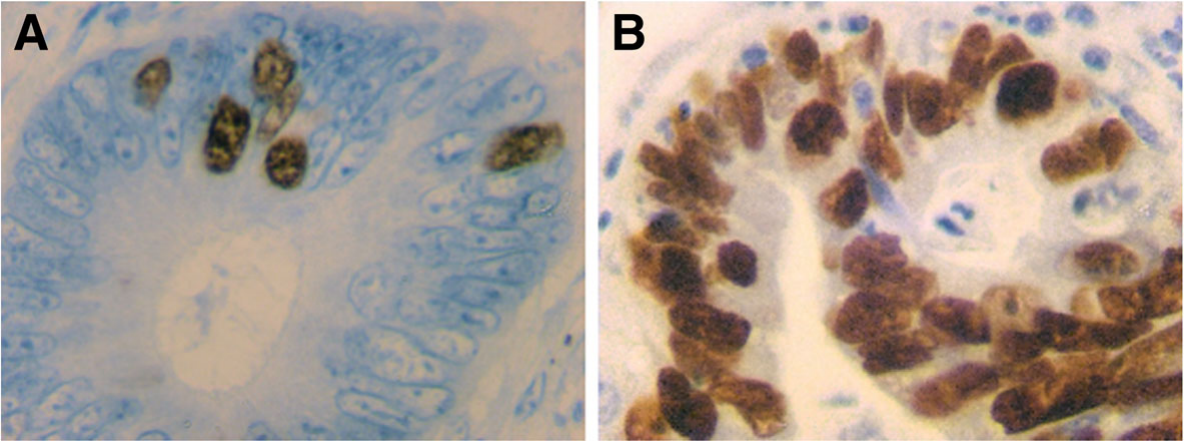
Topoisomerase IIα immunohistochemical staining of colorectal tissue, ABC method × 400 magnification. **a** with a low (< 50%) expression level and (**b**) with high (≥ 50%) expression levels

A quantity of 430 colorectal tissue specimens was procured for evaluation of the Topo II α expression profile. The mean and median expression rate of the entire cohort was 52 and 53.8%. The upper-limit of Topo II α expression was set at 50%. In 267 cases, the degree of expression was ≥50%. An example of Topo II α expression is displayed in the Fig. 1 a + b. Patients aged ≥65 years displayed a significantly lower expression of Topo II α (*p* = 0.005). In the assessment of UICC stages, patients with locally advanced disease (UICC III) had a lower expression of Topo II α compared to patients in early tumour stages (UICC I + II) (*p* = 0.029). Interestingly, the histological grading did not show any coherence to the expression of Topo II α. In terms of histological entities, the adeno carcinoma displayed higher expression profiles than other entities (*p* = 0.041). All data is presented in Additional file 4: Table S1.

### Coherence between topoisomerase II α expression and patient outcome

**Fig. 2:**
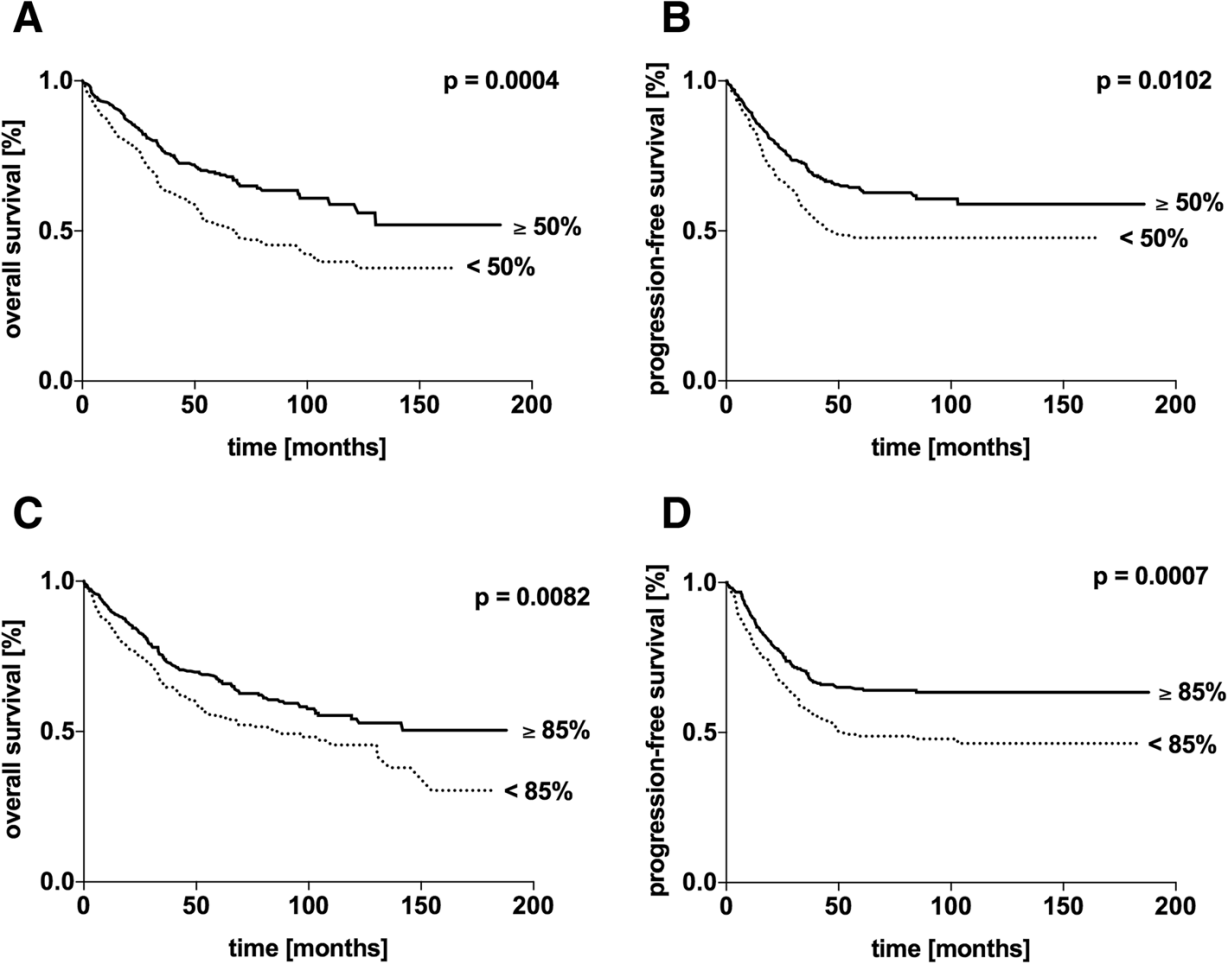
Kaplan-Meier survival analysis of the cumulative overall survival (**a**, **c**) and progression-free survival (**b**, **d**) of patients with colorectal carcinoma stratified by the expression of topoisomerase II α (**a, b**) and minichromosome maintenance protein 6 (**c, d**) according to the cut off. *P*-values were calculated by Log rank tests

**Fig. 3:**
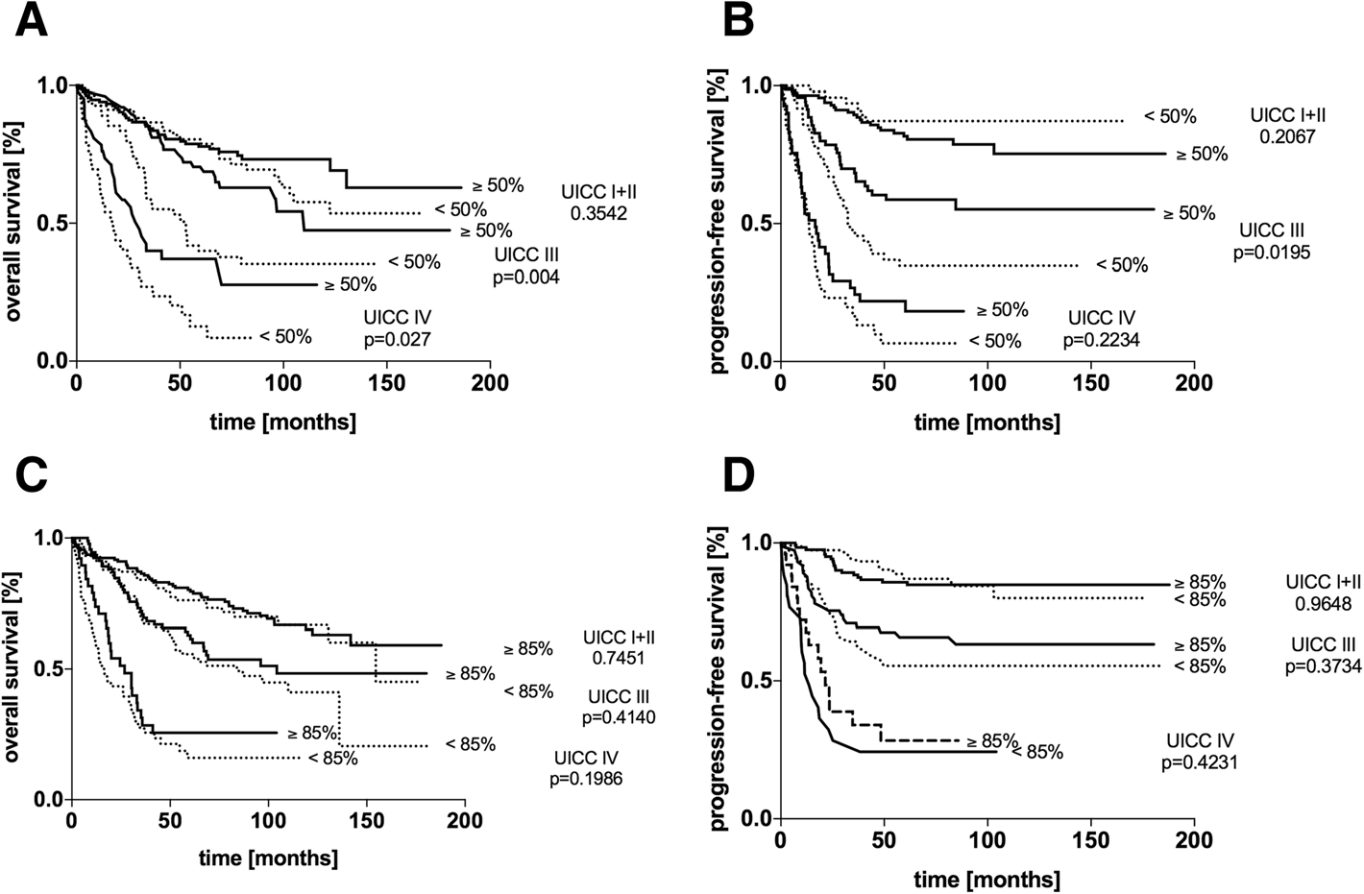
Kaplan-Meier survival analysis of the cumulative overall survival (**a, c**) and progression-free survival (**b, d**) of patients with colorectal carcinoma stratified by the UICC stages I + II, III and IV. Each subset was analysed in respect to topoisomerase II α (**a, b**) and minichromosome maintenance protein 6 expression (**c, d**). P-values were calculated by Log rank tests

In general, low expression rates of Topo II α cohered with a significantly unfavourable outcome (*p* = 0.010) (Fig. 2 a + b). The entire cohort was further analysed by differentiating UICC subsets. The subgroups UICC I + II, UICC III and UICC IV were identified. Within the subset of UICC I + II no difference in the OS or PFS could be monitored (*p* = 0.354 and *p* = 0.207). In the clinically relevant subset of UICC III patients in OS and PFS, low expression rates of Topo II α was a significant negative prognostic marker (*p* = 0.004 and *p* = 0.020). Within the subcategory of UICC IV patients, Topo II α expression was only significantly relevant in the OS (*p* = 0.027) (Fig. 3 a + b).

Regarding histological grading, Topo II α expression showed a significant effect on the patient OS within G2 tumours (*p* < 0.001) (Table 2).

**Table 2:** IHC expression of topoisomerase II a and correlation to the patients´ outcome

	N	OS [months]			DFS [months]		
		expression			expression		
		> 50%	≤50%	p	> 50%	≤50%	p
all	430	n.a.	68.4	**< 0.001**	n.a.	48.4	**0.010**
age (years)							
< 65	211	n.a.	95.7	**< 0.001**	n.a.	n.a.	0.111
≥ 65	215	110.1	53.6	0.188	n.a.	n.a.	0.959
tumor site							
right colon	115	122.6	58.9	0.313	n.a.	n.a.	0.197
left colon + rectum	298	n.a.	68.4	**< 0.001**	n.a.	n.a.	0.797
UICC							
I + II	217	n.a.	n.a.	0.354	n.a.	n.a.	0.207
III	133	110.1	51.9	**0.004**	n.a.	32.4	**0.020**
IV	76	30.2	17.6	**0.027**	16.6	13.6	0.223
histological grading							
I	9	n.a.	n.a.	n.a.	n.a.	n.a.	0.480
II	339	n.a.	66.1	**< 0.001**	n.a.	n.a.	0.283
III	67	98.6.	28.8	0.207	n.a.	n.a.	0.390
histology							
adeno carcinoma	364	n.a.	68.4	**< 0.001**	n.a.	n.a.	0.259
mucinous + signet-ring carcinoma	56	59.1	53.6	0.881	n.a.	n.a.	0.923
resection margin							
R0	404	n.a.	58.9	**0.009**	n.a.	n.a.	0.299
R1 + R2	18	58.0	68.4	0.168	54.4	47.8	0.797

All *P* values in bold, are regarded as statistically significant. Abbreviations: *UICC* Union internationale contre le cancer, *n.a.* not achieved

Analysing the entire cohort and setting the cut-off for Topo II α expression at 50%, patients above the upper-limit had a highly significant beneficial outcome (*p* < 0.001) with a median OS of 69.2%, in comparison to an OS of 52.9% in the subset of < 50% expression of Topo II α. Analogue to the above-mentioned findings a high focal expression of Topo II α (high quantity in hotspots) was correlated with a significant beneficial outcome (*p* = 0.004) (Additional file 2: Figure S2 A).

### Expression of minichromosome maintenance protein 6 correlated to clinic-pathological characteristics

**Fig. 4:**
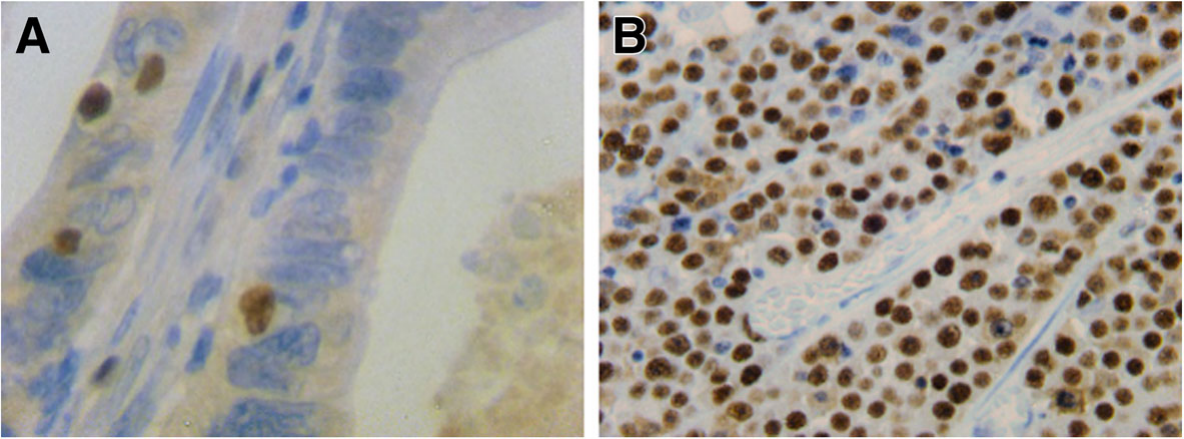
Minichromosome Maintenance Protein 6 immunohistochemical staining of colorectal tissue, ABC method × 400 magnification. **a** with a low (< 85%) expression level and (**b**) with high (≥ 85%) expression levels

A total of 570 tissue specimens were analysed regarding MCMC6. The median expression was 85.8% while the mean expression was 82.8% (range 97.0–27.6%). Based on these findings, the cut-off value of MCM6 expression was set at 85%. In 306 (53.7%) cases, the expression was ≥85% and in 264 cases (46.3%) the expression levels were < 85%. An example of MCM6 expression is displayed in Fig. 4 A + B. Classifying the cohort by UICC stages, advanced tumour stages (III + IV) displayed significantly less expression of MCM compared to locally confined tumours (*p* = 0.012 and *p* < 0.001). In terms of histological grading, significantly less MCM6 expression levels were observed in higher differentiated tumours. There was no statistically significant coherence between patient age and the degree of MCM6 expression. All data is presented in Additional file 5: Table S2.

### Coherence of minichromosome maintenance protein 6 expression and patient outcome

The group of patients that were diagnosed with colorectal neoplasm and MCM6 expression levels below 85%, a significantly poor OS (*p* = 0.008) and PFS (*p* < 0.001) were monitored in the univariate analysis (Fig. 2 c + d). Stratifying the cohort by means of the UICC staging, different to Topo II α expression, MCM6 expression did not correlate with a statistically poor OS or PFS in any of the UICC-subgroups (Fig. 3 c + d). However, categorizing by age groups, in young patients (< 65 years), MCM6 expression levels were linked significantly to a poorer outcome. Regarding histological grading, within the subset of G2 patients, an expression rate above 85% was linked to a significantly poorer outcome (Table 3). Similar to the focal Topo II α expression, MCM6 hotspots were correlated to a poor patient outcome (*p* = 0.013) (Additional file 2: Figure S2 B).

**Table 3:** IHC expression of minichromosome maintenance protein 6 and correlation to the patients´ outcome

	N	OS [months]			DFS [months]		
		expression			expression		
		> 85%	≤85%	p	> 85%	≤85%	p
all	570	n.a.	87.0	**0.008**	n.a.	51.0	**0.001**
age (years)							
< 65	270	n.a.	136.0	**0.007**	n.a.	n.a.	0.418
≥ 65	280	82.0	51.9	0.246	n.a.	n.a.	0.419
tumor site							
right colon	162	104.3	49.8	**0.026**	n.a.	n.a.	0.135
left colon + rectum	385	n.a.	110.1	0.086	n.a.	n.a.	0.658
UICC							
I + II	262	154.6	154.6	0.745	n.a.	n.a.	0.965
III	184	84.0	84.0	0.414	n.a.	n.a.	0.373
IV	101	16.4	16.4	0.199	13.6	21.5	0.423
histological grading							
I	9	n.a.	n.a.	n.a.	n.a.	n.a.	n.a.
II	448	n.a.	104.9	**0.008**	n.a.	n.a.	0.149
III	92	104.3	33.7	0.393	n.a.	n.a.	0.839
histology							
adeno carcinoma	466	n.a.	104.9	**0.026**	n.a.	n.a.	0.837
mucinous + signet-ring carcinoma	75	11.3	41.9	0.103	n.a.	34.7	**0.006**
resection margin							
R0	509	n.a.	130.5	0.115	n.a.	n.a.	0.244
R1 + R2	29	11,3	15.3	0.308	9,7	n.a.	**0.001**

All *P* values in bold, are regarded as statistically significant. Abbreviations: UICC – Union internationale contre le cancer; n.a. – not achieved

### Comparison of MCM6 and topo II α expression levels

In the entire cohort, MCM6 expression was significantly higher (mean 82.8%) than the expression of Topo II α (mean 52.0%) (*p* < 0.001) (Additional file 3: Figure S3 A). Furthermore, a significant correlation (r = 0.433, *p* < 0.001) between the expression of both proliferative markers was observed (Additional file 3: Figure S3 B). Corresponding to this, the analysis of hot spots was significantly higher in MCM6 than Topo II α (*p* < 0.001) (Additional file 3: Figure S3 C).

## Conclusions

Colorectal carcinoma is a major tumour entity and is accountable for the second greatest cause of death in tumour patients [19]. In assessment of the prognosis, prognostic markers are required in addition to the UICC-staging. Dysfunctional cell proliferation plays a key role in neoplasms. Evaluation of proliferative markers in the routine diagnosis of carcinomas is essential. For example, IHC of the proliferative marker Ki-67 is well accepted and executed on a regular basis. High levels of Ki-67 expression indicate rapid tumour growth and are associated with a poor clinical outcome [20–23]. Regarding colorectal carcinoma, contradictory conclusions concerning the proliferation markers have been made. Multiple studies described high expression levels of Ki-67 to be a negative prognostic marker [23–25], whereas other studies came to the opposite conclusion [26]. A few studies did not monitor any impact of the Ki-67 expression levels on the clinical outcome [27].

In this study we focused on two key player proteins in cell division, the topoisomerase II α (Topo II α) and minichromosome maintenance protein 6 (MCM6). We here applied IHC of Topo II α and MCM6 to a large and representative cohort of patients diagnosed with colorectal carcinoma.

IHC analysis was performed in order to detect the expression levels of Topo II α using the primary antibody Ki-S4, developed in the Institute of Haematopatholgy at the University Hospital Kiel. The antibody was proven to be a specific marker for Topo II α [8]. The expression of Topo II α was previously shown to be a significant prognostic indicator in breast cancer and mantel cell lymphoma, where a high intensity of expression was linked to a poor clinical outcome [5, 28]. Data of IHC for the detection of Topo II α expression in large and representative cohorts of CRC patients are limited. Boonsong et al. performed IHC to detect Topoisomerase I levels in 249 CRC patients but was unable to find a correlation neither to histo-pathological characteristics, nor to OS [29]. However, another recent study does reveal a significant correlation in terms of prolonged DFS and OS in patients with high expression rates of Topoisomerase I [30]. Our analysis also revealed a highly significant correlation between the Topo II α expression and the OS and DFS. Synoptically, our data is partially contradictory to previous studies. Lacking analysis of Topo II α in reasonably sized cohorts of patients suffering of CRC, validation is critical. Regarding the patient age, a significant coherence to Topo II α expression was monitored. In young patients (≤65 years), the expression was significantly higher which is likewise a contrary result to the study of Boonsong et al. [29]. Furthermore, within the cohort of younger patients, we were able to identify Topo II α expression as a prognostic marker. High expression rates cohered with a beneficial clinical outcome. As to why the prognostic value is only in the subset of young patients must be further explored – experimental validation is currently lacking.

CRC localised at the right hemi colon is generally associated with an inferior prognosis, we therefore expected expression levels of Topo II α to be significantly lower. To our surprise, the locus of neoplasia (left vs right hemi colon) did not prove any difference in expression rates of Topo II α. Patients diagnosed with an adeno carcinoma and Topo II α expression levels above the cut-off showed a highly significant favourable outcome.

Locally advanced tumour progression is accompanied with lower rates of Topo II α expression. Comparing UICC I + II with UICC III, a significant decrease in expression was monitored. Between UICC III and UICC IV, no difference was asserted. Within each UICC stage, significant impact of Topo II α expression levels on the clinical outcome was observed. These findings prove the prognostic impact of assessing Topo II α expression levels using IHC. In conclusion, our data provides an additional tool to the UICC classification in terms of prognosis and clinical outcome to identify Patients at risk, which may be of benefit to an (neo-) adjuvant treatment.

In further analysis, we assessed the expression levels of MCM6 and clinical characteristics, as well as patient outcome. CRC tissue specimens of a large cohort of patients were studied using IHC with the primary antibody Ki-MCM6, that is highly specific to the MCM6. The relevance of MCM in malignancies has been affirmed in various studies [15–17, 31]. An analysis of MCM6 in patients diagnosed with CRC was absent.

As expected, the mean expression level of MCM6 (83%) was significantly higher than with Topo II α (52%). MCM6 is involved in the early phase of cell cycle replication. The protein is partly involved in the G_1_ phase. Hence, a larger quantity of cells (including cells in early stages of the cell cycle) is stained by IHC [13]. The above-mentioned finding may explain the different quantity of expression when comparing Topo II α with MCM6. Similar results have been demonstrated in other tumour entities [6]. Correlation of Topo II α and MCM6 was clearly demonstrated. Neoplastic tissue with low expression levels of MCM6 exhibited low levels of Topo II α expression.

We did not expect that MCM6 expression levels would negatively correlate with the UICC staging. In progressive tumours, lower expression levels of MCM6 were observed, which is contrary to the Topo II α expression levels in our cohort. We expected high levels of MCM6 in advanced tumours with rapid tumour growth and subsequent greater cell proliferation as previously described by Giaginis et al. in terms of MCM2 expression [32].

Concerning the OS and DFS, expression levels above the cut-off were associated with a favourable outcome. Furthermore, in young patients (≤65 years) with histologically graded G2 adeno carcinoma, MCM6 expression levels above the cut-off also demonstrated a significant marker for a beneficial outcome.

For the first time our study presents data of Topo II α and MCM6 IHC detected expression levels in a large representative cohort of patients diagnosed with CRC. Contrary to the expected outcome, high expression levels of the proliferative markers MCM6 and Topo II α represent a significantly negative prognostic marker.

Increased cell proliferation was generally thought to be responsible for tumour progression and metastasizing. Whereby, as suggested by our data, rather poorly differentiated tumours with scarce cell proliferation seem to be liable for a poor progression of the disease.

In summary, we propose that from a prognostic point of view, high proliferative cell turnover should not be equated with a poor histological tumour differentiation. We finally conclude that assessing the proliferative turnover could be used for risk stratification of CRC patients in the future. Undoubtedly, our data is controversial in context of other malignancies, but carcinomas are diverse, and should not all be investigated in analogy. In this MS we present genuine data exhibiting novel findings in MCM6 and Topo II alpha exploration, that truthfully cannot be elucidated in any manner. A more in-depth investigation is required in order to demonstrate and consolidate our findings in validation cohorts.

## Additional files

Additional file 1: **Figure S1 A + B.** Kaplan-Meier analysis of the cumulative overall (A) and progression-free (B) survival of patients with a colorectal carcinoma and staged according to the UICC classification. The *p*-value was calculated by log-rank test. (TIFF 398 kb)

Additional file 2: **Figure S2 A + B.** Kaplan-Meier analysis of the cumulative overall survival of patients with a colorectal carcinoma and stratified by the characteristic of hotspots of (A) topoisomerase II alpha and (B) minichromosome maintenance protein 6 expression. The occurrence of hotspots significantly correlates with a worse patients ´ outcome. The *p*-value was calculated by log-rank test. (TIFF 488 kb)

Additional file 3: **Figure S3 A-C.** (A) Expression levels of topoisomerase II alpha and minichromosome maintenance protein 6. (B) Significant correlation (r = 0.433, *p* < 0.001) between both proliferative markers. (C) Frequency of hot spots within the entire cohort. (TIFF 521 kb)

Additional file 4: **Table S1.** Coherence of topoisomerase II alpha IHC expression to clinical and histological criteria. (XLSX 9 kb)

Additional file 5: **Table S2.** Coherence of minichromosome maintenance protein 6 IHC expression to clinical and histological criteria. (XLSX 9 kb)

## Abbreviations

CRC: Colorectal cancer
FAP: Familial adenomatous polyposis
FISH: Fluorescent in situ hybridization
HNPCC: Hereditary nonpolyposis colorectal cancer
MCM6: Minichromosome maintenance protein 6
MMR: Mismatch repair gene systems
MSI: Microsatellite instability
OS: Overall survival
PCNA: Proliferating cell nuclear antigen
PFS: Progression-free survival
TNM: TNM Classification of Malignant Tumours
Topo IIα: Topoisomerase II alpha
UICC: Union for International Cancer Control
