# Supplementary figures and images for: Prognostic relevance of topoisomerase II α and minichromosome maintenance protein 6 expression in colorectal cancer
Source: BMC Cancer. 2019 May 9;19:429. doi: 10.1186/s12885-019-5631-3 (PMC6507179; doi:10.1186/s12885-019-5631-3)

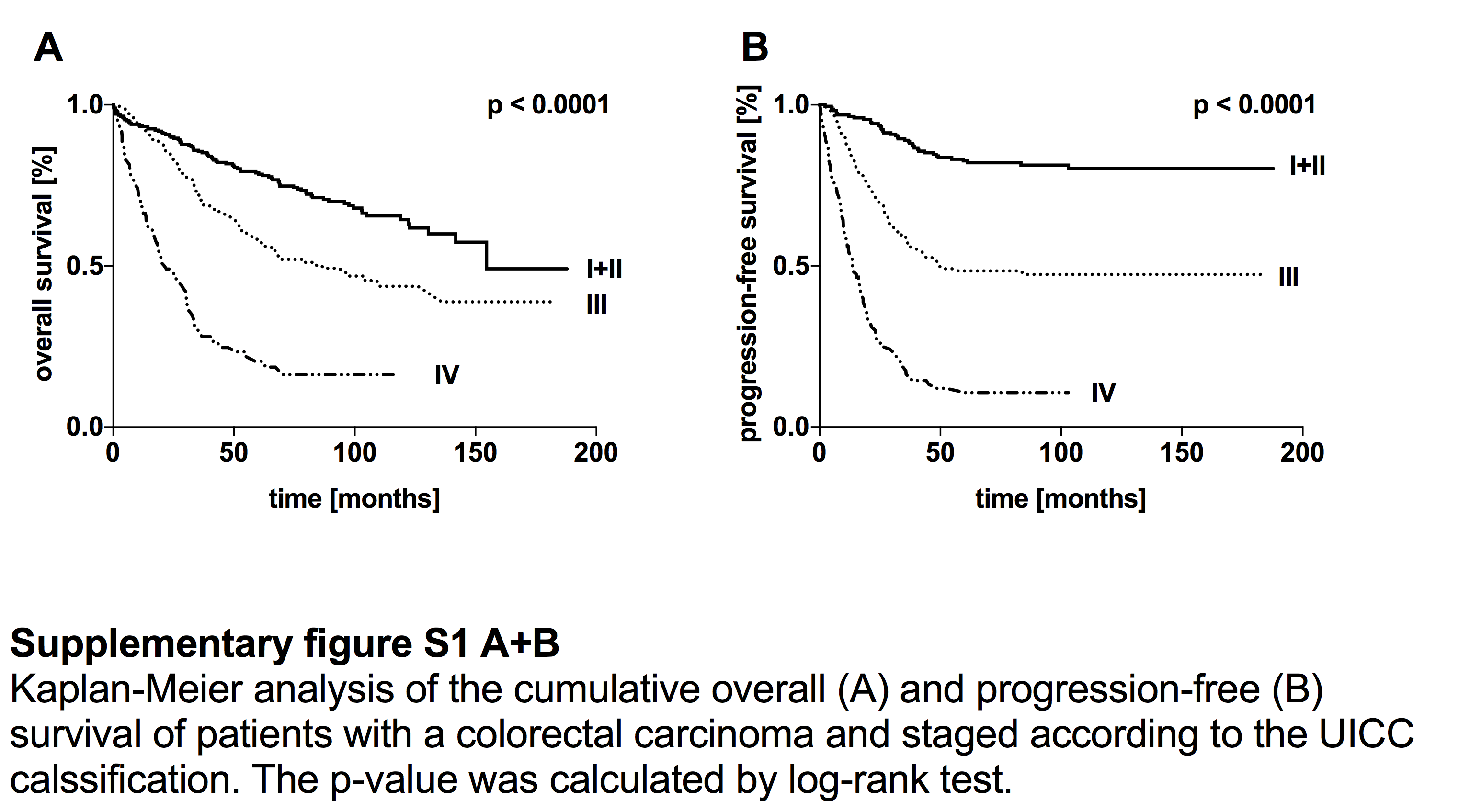

Supplement: Supplementary file 1 — Figure S1 A + B. Kaplan-Meier analysis of the cumulative overall (A) and progression-free (B) survival of patients with a colorectal carcinoma and staged according to the UICC classification. The p-value was calculated by log-rank test. (TIFF 398 kb) [file 12885_2019_5631_MOESM1_ESM.tiff]

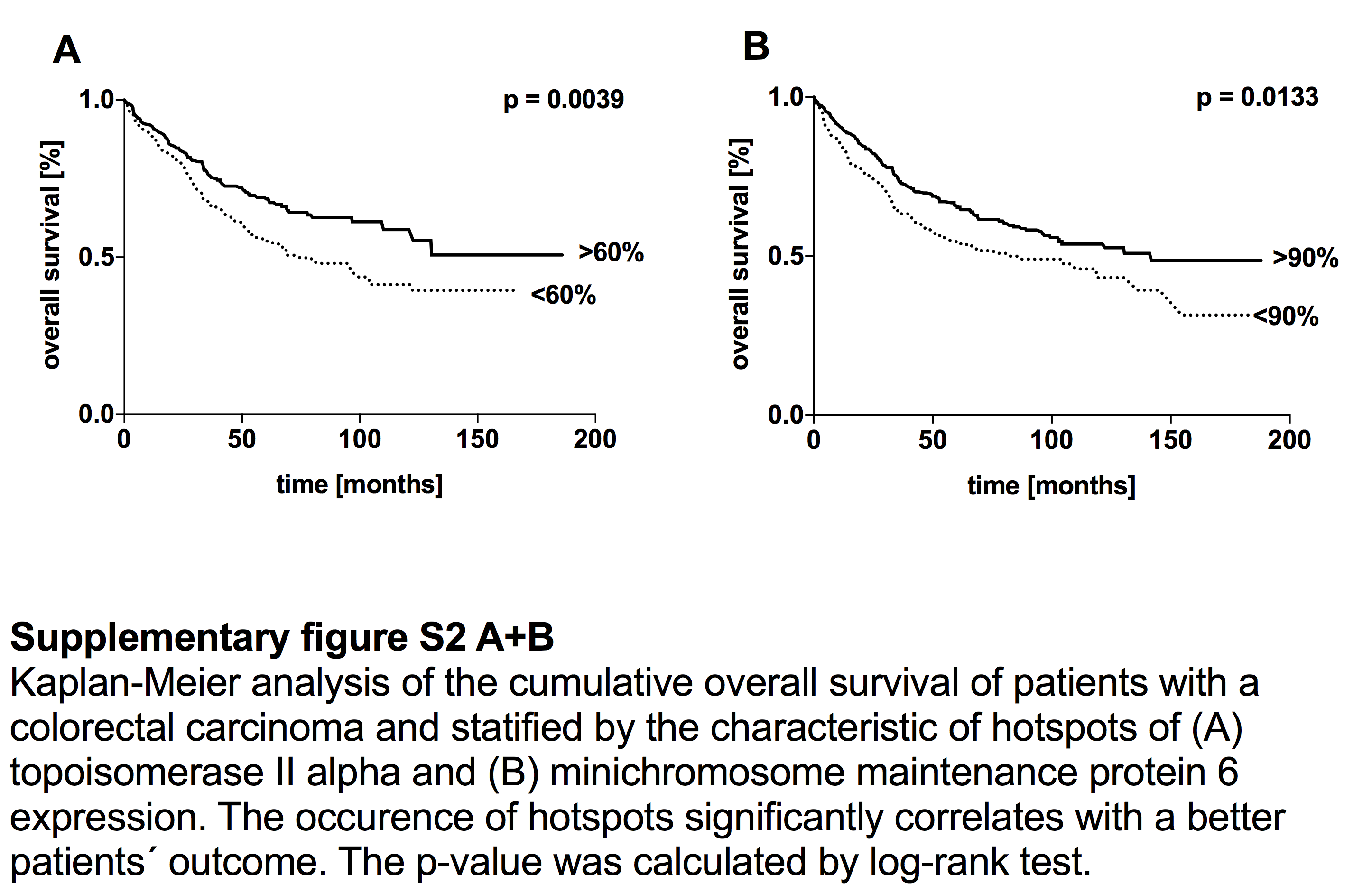

Supplement: Supplementary file 2 — Figure S2 A + B. Kaplan-Meier analysis of the cumulative overall survival of patients with a colorectal carcinoma and stratified by the characteristic of hotspots of (A) topoisomerase II alpha and (B) minichromosome maintenance protein 6 expression. The occurrence of hotspots significantly correlates with a worse patients ´ outcome. The p-value was calculated by log-rank test. (TIFF 488 kb) [file 12885_2019_5631_MOESM2_ESM.tiff]

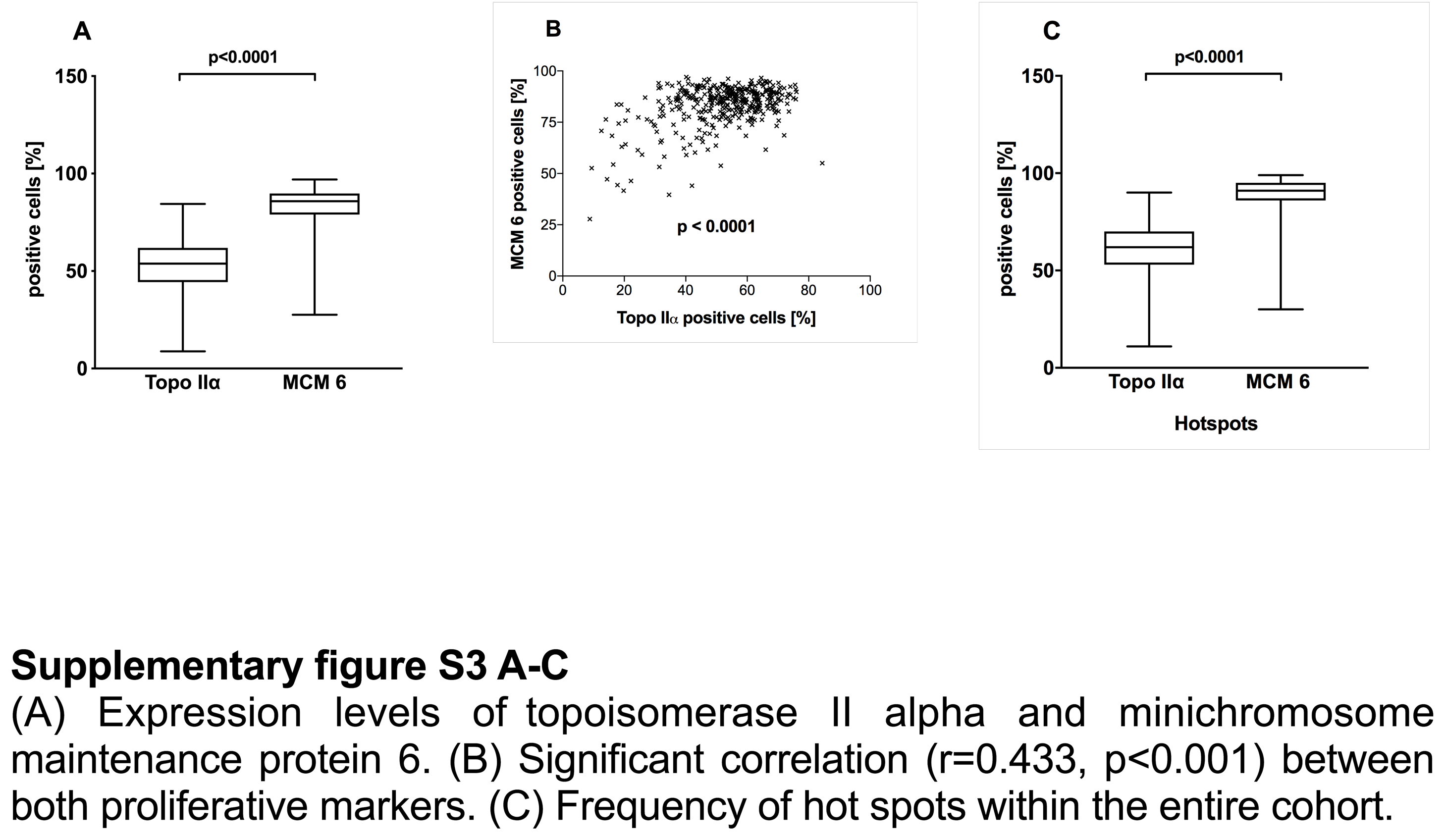

Supplement: Supplementary file 3 — Figure S3 A-C. (A) Expression levels of topoisomerase II alpha and minichromosome maintenance protein 6. (B) Significant correlation (r = 0.433, p < 0.001) between both proliferative markers. (C) Frequency of hot spots within the entire cohort. (TIFF 521 kb) [file 12885_2019_5631_MOESM3_ESM.tiff]
